# Association between different obesity patterns and the risk of NAFLD detected by transient elastography: a cross-sectional study

**DOI:** 10.1186/s12876-024-03303-x

**Published:** 2024-07-10

**Authors:** Jingjing Sun, Chun Yan, Jing Wen, Fang Wang, Han Wu, Fang Xu

**Affiliations:** Department of Ultrasound, Shanghai Fourth People’s HospitalTongji University School of MedicineHongkou District, No. 1279, Sanmen Road, Shanghai, 200434 China

**Keywords:** Obesity patterns, Transient elastography, NAFLD, Hepatic fibrosis, Cross-sectional

## Abstract

**Background:**

Obesity has become a major global public health challenge. Studies examining the associations between different obesity patterns and the risk of nonalcoholic fatty liver disease (NAFLD) are limited. This study aimed to investigate the relationships between different obesity patterns and the risk of NAFLD in a large male population in the US.

**Methods:**

Data from the 2017 to March 2020 National Health and Nutrition Examination Survey (NHANES) were utilized. Liver steatosis and fibrosis were assessed with FibroScan using the controlled attenuation parameter (CAP) and liver stiffness measurements (LSM). Steatosis was identified with a CAP value of 248 dB/m or higher. Abdominal obesity was defined by a waist circumference (WC) of 102 cm or more for males and 88 cm or more for females. Overweight was defined as a body mass index (BMI) of 24.0 kg/m^2^ and above. General obesity was identified with a BMI of 28.0 kg/m^2^ or higher. Obesity status was categorized into four types: overweight, general obesity, abdominal obesity, and combined obesity. Multivariate logistic regression, adjusting for potential confounders, was used to examine the link between obesity patterns and NAFLD risk. Subgroup analysis further explored these associations.

**Results:**

A total of 5,858 adults were included. After multivariable adjustment, compared to the normal weight group, the odds ratios (ORs) [95% confidence interval (CI)] for NAFLD in individuals with overweight, general obesity, abdominal obesity, and combined obesity were 6.90 [3.74–12.70], 2.84 [2.38–3.39], 3.02 [2.02–4.51], and 9.53 [7.79–11.64], respectively. Subgroup analysis showed the effect of different obesity patterns on NAFLD risk was stable among individuals with different clinical conditions. In the fully adjusted multivariate logistic regression model, WC was positively associated with NAFLD risk (OR: 1.48; 95% CI: 1.42–1.53; P < 0.001). WC also demonstrated strong discriminatory ability for NAFLD in Receiver Operating Characteristic (ROC) analysis, achieving an Area Under the Curve (AUC) of 0.802.

**Conclusions:**

Different patterns of obesity are risk factors for NAFLD. An increase in WC significantly increased NAFLD risk. More attention should be paid to preventing different patterns of obesity among adults.

**Supplementary Information:**

The online version contains supplementary material available at 10.1186/s12876-024-03303-x.

## Background

Hepatic steatosis, an accumulation of fat in the liver that is usually linked to obesity, can proceed to fibrosis, cirrhosis, and nonalcoholic fatty liver disease (NAFLD) [1]. Given the rising incidence of obesity globally, the deleterious effects hepatic steatosis is becoming a growing challenge for public health. NAFLD is characterized by the accumulation of fat in the liver without secondary causes such as extensive alcohol intake and viral infections [2, 3]. NAFLD is now the most common type of hepatic steatosis, and it is estimated that 32% of adults have NAFLD, with a higher prevalence in males (40%) compared to females (26%) [4]. It is regarded as a hepatic manifestation of metabolic syndrome and is associated with obesity, dyslipidemia, diabetes, and hypertension [5]. Although significant efforts have focused on NAFLD drug development, treatment generally remains limited to lifestyle modification [6], highlighting the critical need for a deeper understanding of its pathogenesis and risk factors.

Ultrasound examination is the most commonly used imaging method for the diagnosis of NAFLD, as it is a non-invasive, easily accessible, and cost-effective tool that can identify changes in liver texture associated with fat accumulation [7]. This method has high clinical relevance because it allows for early detection and ongoing monitoring of disease progression, which is essential for managing NAFLD effectively. This emphasizes the role of regular liver health assessments, particularly in populations at risk due to obesity or metabolic syndrome.

Obesity has emerged as a significant public health issue, linked to various complications, including hyperlipidemia, hypertension, arteriosclerosis and NAFLD [8, 9]. Obesity is a condition characterized by excessive body fat accumulation that poses a risk to health, and it is a significant risk factor for the development of NAFLD [10]. The established link between obesity and NAFLD, driven by insulin resistance, metabolic dysregulation, and systemic inflammation [11, 12], points to the complex interplay of genetic, environmental, and lifestyle factors in the development of these conditions. Prevention and management strategies for both conditions often overlap and include lifestyle modifications such as diet, physical activity, and weight loss.

The classification of obesity is based on the pattern of fat distribution. Body mass index (BMI) is a common indicator of general obesity. However, BMI fails to accurately measure the extent of abdominal fat obesity [13]. In fact, fat distribution may be an important factor in the development of NAFLD [14]. Waist circumference (WC) can be used to determine the status of abdominal fat accumulation and is often used to define abdominal obesity. It has been confirmed that WC can complement BMI to assess abdominal obesity. Thus, the combination of BMI and WC might be better to evaluate the fat distribution. However, studies on the relationship between the combination of BMI and WC and NAFLD risk are limited. Therefore, it is necessary to investigate the association between the combination of BMI and WC and NAFLD in a large population using data from the National Health and Nutrition Examination Survey (NHANES).

## Methods

### Source Population

NHANES is an ongoing survey conducted every two years in the United States to assess the health and nutrition of both adults and children. Using a detailed, multi-stage sampling method, it provides valuable insights into the nation's health trends. The survey collects a wide range of data, including demographic details, questionnaire responses, examination findings, and laboratory test results. For this particular study, data from the 2017 to 2020 cycles were used, as they included specific liver transient elastography information. This data is publicly accessible through the NHANES website at https://wwwn.cdc.gov/nchs/nhanes/Default.aspx.

**Fig. 1:**
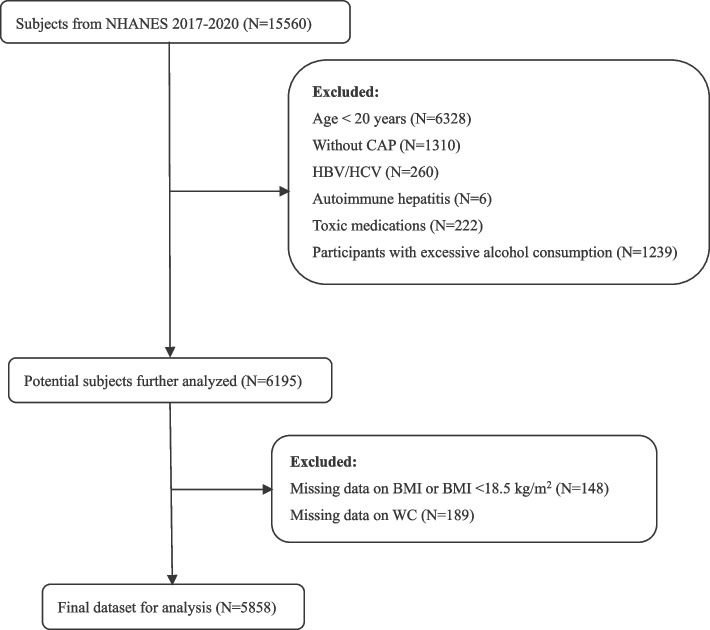
Flowchart for the selection of the participants of the present study

A total of 15,560 participants were enrolled in this cycle. We focused on adult participants who, at the time of their transient elastography scan, had reliable FibroScan® readings. These readings required more than 10 measurements and an interquartile range of less than 30% of the median value. The Controlled Attenuation Parameter (CAP), measured by transient elastography, serves as an indicator of the extent of fatty buildup in the liver, with higher CAP values signifying more severe hepatic steatosis. NAFLD was identified by a CAP score of 248 dB/m or higher, a threshold established from a comprehensive meta-analysis that evaluated CAP scores for diagnosing NAFLD [15]. LSM ≥ 8.0 kPa was used as a cutoff suggesting clinically relevant fibrosis [16]. We excluded individuals with alternative liver disease causes, such as being positive for hepatitis B surface antigen, hepatitis C antibody, autoimmune hepatitis, or consuming excessive alcohol (over 3 daily drinks for women and 4 for men). We also excluded medications that cause steatosis, such as amiodarone, methotrexate, corticosteroids, tamoxifen. Additional exclusions were for those lacking BMI data or having a BMI under 18.5 kg/m^2^, and those missing WC information. As a result, 5858 participants met all the inclusion criteria and were analyzed in the study (Fig. 1).

### Anthropometric measurements

Trained professionals carried out measurements of weight, height, and WC using standard methods and devices. These measurements were taken at the Mobile Examination Center (MEC) by competent health technicians who were regularly evaluated through direct observation, review of data, and feedback from expert examiners. Participants were measured while wearing light clothing and no shoes. WC, used to determine abdominal obesity, was recorded just above the hip bones and divided into four groups. Body Mass Index (BMI) was used to assess overall obesity, calculated by dividing a person's weight in kilograms by their height in meters squared (kg/m^2^). Detailed procedures for all anthropometric measurements are available on the NHANES website.

### Different obesity patterns

Based on the guidelines from the International Diabetes Federation (IDF), a normal weight is characterized by a BMI ranging from 18.5 kg/m^2^ to 23.9 kg/m^2^. A BMI of 24.0 kg/m^2^ and above is considered overweight. General obesity is identified when a person's BMI is 28.0 kg/m^2^ or higher, provided the WC is not abnormally large. Abdominal obesity is defined by a WC of 102 cm or more for males and 88 cm or more for females, while also maintaining a BMI within the normal range. When an individual exhibits both general and abdominal obesity, it is classified as compound obesity [17, 18]. As a result, obesity is primarily categorized into four distinct types: overweight, general obesity, abdominal obesity and compound obesity [19].

### Covariates of interest

In this study, we included various factors such as age, gender, race/ethnicity, education level, BMI, WC, Controlled Attenuation Parameter (CAP), liver stiffness (measured in kilopascals or kPa), and medical conditions including diabetes, high blood pressure, cancer, and cardiovascular disease (CVD). Ethnicity was grouped into Mexican–American, non-Hispanic white, non-Hispanic black, and other. Smoking habits were classified based on participants' self-reports as current smokers, former smokers, or those who have never smoked. The presence of statins, antihypertensive drugs, and antidiabetic agents was based on the participants’ responses on the NHANES medical conditions questionnaire. Individuals were considered to have diabetes if they were previously diagnosed by a doctor or had a fasting blood sugar level of 126 mg/dL or higher. Those identified as having hypertension had either been previously diagnosed, were on medication for high blood pressure, or had blood pressure readings of 140/90 mmHg or higher. CVD was defined as having a history of heart-related diseases like congestive heart failure, coronary artery disease, angina, or myocardial infarction. Blood samples were collected and used to examine the levels of total cholesterol, high-density lipoprotein cholesterol (HDL-C), aspartate aminotransferase (ALT, IU/L) and aspartate transaminase (AST, IU/L), gamma glutamyl transferase (GGT, U/L), alkaline phosphatase (ALP, U/L), total bilirubin, and uric acid. The NHANES website details the protocols for these biochemical tests.

### Statistical analysis

Continuous variables were expressed as mean ± standard deviation (SD) or the median (interquartile range) and categorical variables as number (percentage). We assessed the initial characteristics of participants with various obesity patterns using statistical tests. For categorical variables, we employed the Chi-square test to identify differences. For continuous variables, we applied the one-way ANOVA test when the data followed a normal distribution, and the Shapiro–Wilk test was used for data with a skewed distribution. Multivariate logistic regression models were used to evaluate the independent relationship between different obesity patterns and the prevalence of NAFLD. We created three regression models, adjusting for various covariates to minimize the impact of confounding factors. The choice of these covariates was guided by both theoretical considerations and statistical evidence. Model I was adjusted for no covariates. Model II was a minimally-adjusted model adjusted for gender, age, race, and education level. Model III was further adjusted for smoking status, history of CVD, history of malignancy, history of diabetes, history of hypertension, ALT, AST, total bilirubin, ALP, GGT, uric acid, total cholesterol, HDL cholesterol, statins, antihypertensive drugs, and antidiabetic agents. In our analysis, individuals of normal weight served as the reference group, and we calculated the odds ratios (ORs) along with their 95% confidence intervals (CIs) for comparison. We used Restricted Cubic Spline analysis (RCS) with four knots to assess the non-linear relationships between WC and the likelihood of NAFLD. Additionally, we utilized the receiver operating characteristic (ROC) curve to evaluate the effectiveness of WC in distinguishing individuals who have NAFLD. To further investigate the association between obesity patterns and NAFLD across different demographic groups, we conducted stratified analyses by age (< 60 or ≥ 60 years), gender (male or female), CVD (yes or no), diabetes (yes or no), hypertension (yes or no), education level (less than high school, high school, above high school), and race (Mexican–American, non-Hispanic white, non-Hispanic black, and other). There is evidence that being overweight or having obesity increases the risk of various malignancies [20]. Furthermore, to ensure the robustness of our findings, we performed sensitivity analyses by excluding participants with a history of malignancy. All statistical analyses were performed using R version 4.2.1 software (R Foundation for Statistical Computing, Vienna, Austria). A two-sided *p*-value < 0.05 indicated significance for all analyses.

## Results

### The baseline characteristics of participants

Our study included 5858 participants, with 2827 males and 3031 females. The distribution of obesity types was as follows: general obesity in 71 participants (1.21%), overweight in 1943 participants (33.17%), abdominal obesity in 143 participants (2.44%), and compound obesity in 2400 participants (40.97%). The incidence of NAFLD varied across the groups, with 310 cases (23.83%) in those of normal weight, 54 cases (76.06%) in the general obesity group, 1103 cases (56.77%) in the overweight group, 73 cases (51.05%) in abdominal obesity group, and 1953 cases (81.38%) in the compound obesity group. There were notable differences across these groups in all variables included, as detailed in Table 1.

**Table 1:** Comparison of participants’ characteristics among different obesity patterns

	Normal weight	General Obesity	Overweight	Abdominal obesity	Compound obesity	*P*-value
N	1301	71	1943	143	2400	
BMI (kg/m²)	22.30 ± 1.71	31.09 ± 1.06	27.40 ± 1.43	23.89 ± 0.81	36.57 ± 6.01	<0.001
Waist circumference (cm)	82.14 ± 7.06	97.87 ± 3.69	96.26 ± 7.27	92.28 ± 3.54	115.75 ± 12.86	<0.001
CAP (dB/m)	220.00 ± 47.07	275.80 ± 53.73	258.12 ± 52.99	248.20 ± 45.23	299.28 ± 57.50	<0.001
Age (years)	48.24 ± 18.74	38.51 ± 13.63	53.77 ± 16.74	61.41 ± 15.60	52.20 ± 16.10	<0.001
ALT (IU/L)	17.90 ± 11.35	31.70 ± 24.57	21.51 ± 13.81	15.50 ± 6.73	23.44 ± 16.63	<0.001
AST (IU/L)	20.97 ± 11.70	25.48 ± 15.28	21.45 ± 12.23	19.50 ± 5.58	21.00 ± 10.41	0.006
Total Bilirubin (mg/dL)	0.50 ± 0.31	0.49 ± 0.35	0.47 ± 0.28	0.45 ± 0.27	0.42 ± 0.24	<0.001
ALP (U/L)	71.54 ± 23.83	75.61 ± 19.73	77.32 ± 25.35	80.40 ± 26.35	81.25 ± 24.49	<0.001
GGT (U/L)	24.22 ± 35.50	31.28 ± 17.30	31.12 ± 67.21	23.63 ± 32.39	32.83 ± 37.40	<0.001
Uric acid (mg/dL)	4.86 ± 1.33	5.95 ± 1.11	5.36 ± 1.38	4.68 ± 1.18	5.74 ± 1.51	<0.001
Total cholesterol (mg/dL)	184.10 ± 40.85	187.43 ± 42.54	188.19 ± 40.73	202.43 ± 45.39	184.74 ± 39.46	<0.001
HDL cholesterol (mg/dL)	60.41 ± 16.62	46.03 ± 11.36	53.03 ± 14.61	64.91 ± 19.89	48.73 ± 12.76	<0.001
Alcohol (grams/day)	22.17 ± 9.48	29.92 ± 11.21	23.34 ± 9.63	21.10 ± 7.05	22.68 ± 9.36	<0.001
Gender						<0.001
Female	632 (48.58%)	4 (5.63%)	879 (45.24%)	139 (97.20%)	1377 (57.38%)	
Male	669 (51.42%)	67 (94.37%)	1064 (54.76%)	4 (2.80%)	1023 (42.62%)	
Race						<0.001
Mexican American	87 (6.69%)	12 (16.90%)	227 (11.68%)	12 (8.39%)	274 (11.42%)	
Non-Hispanic Black	292 (22.44%)	24 (33.80%)	447 (23.01%)	29 (20.28%)	813 (33.88%)	
Non-Hispanic White	430 (33.05%)	10 (14.08%)	637 (32.78%)	71 (49.65%)	838 (34.92%)	
Other	492 (37.82%)	25 (35.21%)	632 (32.53%)	31 (21.68%)	475 (19.79%)	
Education						0.024
Less than high school	214 (16.46%)	8 (11.27%)	380 (19.60%)	23 (16.08%)	398 (16.61%)	
High school	279 (21.46%)	19 (26.76%)	419 (21.61%)	33 (23.08%)	596 (24.87%)	
Above high school	807 (62.08%)	44 (61.97%)	1140 (58.79%)	87 (60.84%)	1402 (58.51%)	
Malignancy	116 (8.92%)	1 (1.41%)	230 (11.84%)	23 (16.08%)	254 (10.58%)	0.001
CVD	117 (8.99%)	1 (1.41%)	214 (11.01%)	16 (11.19%)	292 (12.17%)	0.004
Smoking status						<0.001
Never	835 (64.23%)	47 (66.20%)	1234 (63.51%)	95 (66.43%)	1486 (61.94%)	
Former	241 (18.54%)	12 (16.90%)	458 (23.57%)	28 (19.58%)	627 (26.14%)	
Now	224 (17.23%)	12 (16.90%)	251 (12.92%)	20 (13.99%)	286 (11.92%)	
Diabetes	119 (9.15%)	5 (7.04%)	351 (18.06%)	26 (18.18%)	687 (28.62%)	<0.001
Hypertension	286 (22.03%)	20 (28.17%)	719 (37.00%)	58 (41.13%)	1182 (49.27%)	<0.001
Statins	196 (15.08%)	5 (7.04%)	501 (25.82%)	35 (24.48%)	618 (25.79%)	<0.001
Antihypertensive drugs	269 (20.69%)	14 (19.72%)	699 (36.03%)	64 (44.76%)	1061 (44.28%)	<0.001
Antidiabetic agents	95 (7.31%)	3 (4.23%)	266 (13.71%)	16 (11.19%)	497 (20.74%)	<0.001

Normally distributed continuous variables are presented as the mean ± standard deviation; Non-normally distributed continuous variables are presented as the mean [interquartile range]; Categorical variables are presented as the number (percentage)

### Associations of different obesity patterns with NAFLD

In Model I, the associations between various obesity patterns and the risk of NAFLD were significant. Specifically, those with general obesity had 10 times higher odds of NAFLD (OR = 10.15; 95% CI: 5.80–17.77, *P* < 0.001), individuals with overweight had 4.2 times higher odds (OR = 4.20; 95% CI: 3.59–4.91, *P* < 0.001), individuals with abdominal obesity had 3.33 times higher odds (OR = 3.33; 95% CI: 2.35–4.74, *P* < 0.001), and those with compound obesity had nearly 14 times higher odds (OR = 13.97; 95% CI: 11.86–16.45, *P* < 0.001). These associations persisted in a minimally adjusted model that accounted for gender, age, race, and education level. In a fully adjusted model that also considered ALT, AST, total bilirubin, ALP, GGT, uric acid, total cholesterol, HDL cholesterol, malignancy, CVD, smoking status, diabetes, hypertension, and medication use, the odds of NAFLD were 6.9 times higher in the general obesity group (OR = 6.90; 95% CI: 3.74–12.70; *P* < 0.001), 2.84 times higher in those with overweight group (OR = 2.84, 95% CI: 2.38–3.39; *P* < 0.001), 3 times higher in those with abdominal obesity group (OR = 3.02, 95% CI: 2.02–4.51; *P* < 0.001), and 9.53 times higher in those with compound obesity (OR = 9.53, 95% CI: 7.79–11.64; *P* < 0.001) compared to individuals with normal weight (Table 2).

**Table 2:** Multivariate logistic regression of associations between different patterns of obesity and NAFLD risk

	Model I	Model II	Model III
Exposure	OR (95%CI), P	OR (95%CI), P	OR (95%CI), P
Normal Weight	1(Reference)	1(Reference)	1(Reference)
General Obesity	10.15 (5.80, 17.77) <0.0001	11.66 (6.54, 20.81) <0.0001	6.90 (3.74, 12.70) <0.0001
Overweight	4.20 (3.59, 4.91) <0.0001	4.10 (3.49, 4.82) <0.0001	2.84 (2.38, 3.39) <0.0001
Abdominal Obesity	3.33 (2.35, 4.74) <0.0001	3.79 (2.62, 5.49) <0.0001	3.02 (2.02, 4.51) <0.0001
Compound Obesity	13.97 (11.86, 16.45) <0.0001	18.32 (15.33, 21.89) < 0.0001	9.53 (7.79, 11.64) <0.0001

Model I adjust for: None

Model II adjust for: gender, age, race, education level

Model III adjust for: gender, age, race, education level, ALT, AST, total bilirubin, ALP, GGT, uric acid, total cholesterol, HDL cholesterol, malignancy, CVD, smoking status, diabetes, hypertension, statins, antihypertensive drugs, and antidiabetic agents

### Subgroup analysis

**Fig. 2:**
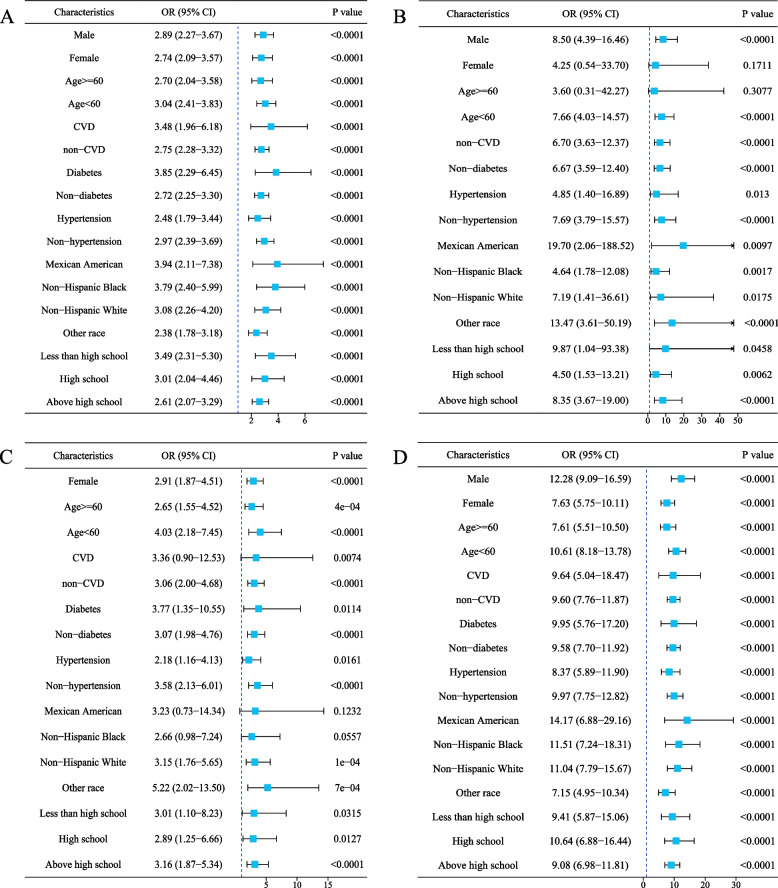
Subgroup analyses for the risks of NAFLD in different obesity pattern groups compared with the normal-weight group. **A** general obesity vs normal-weight group; **B** overweight vs normal-weight group; **C** abdominal obesity vs normal-weight group; **D** compound obesity vs normal-weight group

We performed subgroup analyses stratified by age, gender, race, history of CVD, diabetes, hypertension, and education status to further investigate the association between obesity patterns and the risk of NAFLD across different demographic and clinical subgroups. Our findings indicate that the impact of various obesity patterns on the risk of NAFLD remained consistently significant across the subgroups analyzed. For general obesity (Fig. 2A), the ORs and 95% CIs in key subgroups were as follows: male (OR = 2.89, 95% CI: 2.27–3.67), female (OR = 2.74, 95% CI: 2.09–3.57), age ≥ 60 years (OR = 2.70, 95% CI: 2.04–3.58), age < 60 years (OR = 3.04, 95% CI: 2.41–3.83), non-CVD (OR = 2.75, 95% CI: 2.28–3.32), non-diabetes (OR = 2.72, 95% CI: 2.25–3.30), non-hypertension (OR = 2.97, 95% CI: 2.39–3.69), and non-Hispanic White (OR = 3.08, 95% CI: 2.26–4.20). For overweight (Fig. 2B), the ORs and 95% CIs in key subgroups were: male (OR = 8.50, 95% CI: 4.39–16.46), age < 60 years (OR = 7.66, 95% CI: 4.03–14.57), non-CVD (OR = 6.70, 95% CI: 3.63–12.37), non-diabetes (OR = 6.67, 95% CI: 3.59–12.40), non-hypertension (OR = 7.69, 95% CI: 3.79–15.57), and non-Hispanic White (OR = 7.19, 95% CI: 1.41–36.61). For abdominal obesity (Fig. 2C), the ORs and 95% CIs in key subgroups were: female (OR = 2.91, 95% CI: 1.87–4.51), age < 60 years (OR = 4.03, 95% CI: 2.18–7.45), non-CVD (OR = 3.06, 95% CI: 2.00–4.68), non-diabetes (OR = 3.07, 95% CI: 1.98–4.76), non-hypertension (OR = 3.58, 95% CI: 2.13–6.01), and non-Hispanic White (OR = 3.15, 95% CI: 1.76–5.65). For compound obesity (Fig. 2D), the ORs and 95% CIs in key subgroups were: male (OR = 12.28, 95% CI: 9.09–16.59), female (OR = 7.63, 95% CI: 5.75–10.11), age ≥ 60 years (OR = 7.61, 95% CI: 5.51–10.50), age < 60 years (OR = 10.61, 95% CI: 8.18–13.78), non-CVD (OR = 9.60, 95% CI: 7.76–11.87), non-diabetes (OR = 9.58, 95% CI: 7.70–11.92), non-hypertension (OR = 9.97, 95% CI: 7.75–12.82), and non-Hispanic White (OR = 11.04, 95% CI: 7.79–15.67).

### Association between WC and NAFLD

**Fig. 3:**
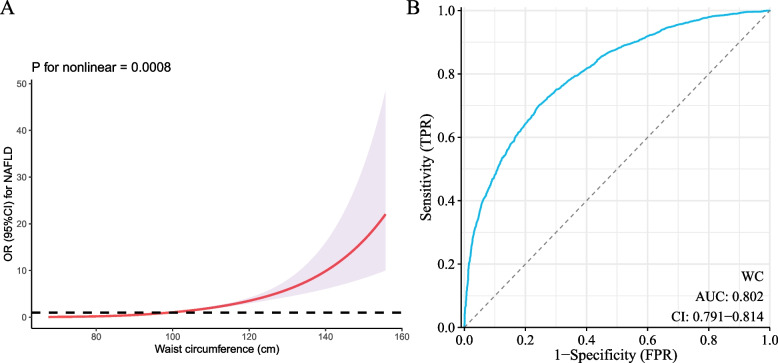
Restricted cubic spline analysis between waist circumference and the risk of NAFLD and the Receiver operating characteristic curve. **A** Restricted cubic spline analysis for the association between waist circumference and the risk of NAFLD; **B** Receiver operating characteristic curve of waist circumference for discriminating NAFLD risk

Given that individuals with abdominal or compound obesity present greater WC and a notably increased rate of NAFLD, we investigated the link between WC and NAFLD. In three different models, an increment in WC by every 10 cm was associated with a higher likelihood of NAFLD: Model I (OR = 2.40; 95% CI: 2.28–2.53; *P* < 0.0001), Model II (OR = 2.51; 95% CI: 2.38–2.66; *P* < 0.0001), and Model III (OR = 2.15; 95% CI: 2.02–2.29; *P* < 0.0001) (Table 3). Even after accounting for factors like gender, age, race, education, liver function tests (ALT, AST, total bilirubin, ALP, GGT), uric acid, lipid levels (total cholesterol, HDL cholesterol), medical history (malignancy, CVD, hypertension, diabetes), lifestyle (smoking), and medication use (statins, blood pressure, and diabetes medications), individuals in the higher quartiles of WC had a greater NAFLD risk. Restricted cubic spline (RCS) analysis also indicated a non-linear positive association between WC and NAFLD prevalence, with risk escalating particularly in the upper quantile (Fig. 3A). The Receiver Operating Characteristic (ROC) analysis further confirmed the robust discriminatory capability of WC in identifying NAFLD risk, evidenced by an Area Under the Curve (AUC) of 0.802 (95% CI: 0.791–0.814), with 96.4 cm pinpointed as the optimal WC cutoff value (Fig. 3B). Furthermore, 623 individuals diagnosed with malignancy were excluded. The main results of the sensitivity analysis did not change significantly (Table S1).

**Table 3:** Multivariate logistic regression model of associations between waist circumference and NAFLD risk

	Model I	Model II	Model III
Exposure	OR (95%CI), P	OR (95%CI), P	OR (95%CI), P
Waist circumference (per 10 cm)	2.40 (2.28, 2.53) <0.0001	2.51 (2.38, 2.66) <0.0001	2.15 (2.02, 2.29) <0.0001
Waist circumference quartiles			
Q1	1(Reference)	1(Reference)	1(Reference)
Q2	3.74 (3.19, 4.39) <0.0001	3.72 (3.15, 4.39) <0.0001	2.74 (2.29, 3.29) <0.0001
Q3	9.01 (7.62, 10.65) <0.0001	9.43 (7.89, 11.27) <0.0001	5.81 (4.77, 7.08) <0.0001
Q4	25.50 (20.85, 31.19) <0.0001	31.25 (25.23, 38.70) <0.0001	16.15 (12.69, 20.56) <0.0001

Model I adjust for: None

Model II adjust for: sex, age, race, education level

Model III adjust for: sex, age, race, education level, ALT, AST, total bilirubin, ALP, GGT, uric acid, total cholesterol, HDL cholesterol; malignancy, CVD, smoking status, diabetes, hypertension, statins, antihypertensive drugs, and antidiabetic agents

## Discussion

This cross-sectional study utilized data from NHANES (2017–2020) to explore the relationship between various obesity patterns and the risk of NAFLD, identified through transient elastography. BMI and WC were used as indicators for general obesity and abdominal obesity, respectively. Our findings highlight the significant association between different obesity patterns—namely, general obesity, overweight, abdominal obesity, and compound obesity—and increased NAFLD risk among U.S. adult population. The likelihood of NAFLD increased significantly with the level of obesity, particularly reaching the highest risk among those with compound obesity. This is consistent with the previous findings, who also reported significant relationships between different obesity patterns and metabolic disorders, emphasizing the role of visceral fat in the onset of such conditions [17, 21].

Most studies have used BMI to evaluate overall obesity. However, BMI alone fails to provide detailed insights into fat distribution across the body and cannot distinguish between fat and muscle [22]. It has been noted that the impact of body fat distribution on the risk of cardiometabolic conditions is more significant than that of BMI [17]. Therefore, it seems that visceral fat plays a major role in the onset of metabolic disorders linked to obesity [23]. The risk of NAFLD is significantly influenced by both BMI and WC, which are indicative of general and abdominal obesity, respectively. Abdominal obesity, which involves an atypical distribution of body fat, has been connected to both insulin resistance and persistent inflammation [24]. Abdominal obesity is a significant risk factor for the development of NAFLD, with its pathophysiological mechanisms primarily attributed to metabolic, inflammatory, and hormonal disturbances [9, 25]. The link between abdominal obesity and NAFLD primarily involves insulin resistance, which is exacerbated by the disproportionate accumulation of visceral fat. This condition disrupts normal fat processing and leads to a higher flow of free fatty acids to the liver [26, 27]. This process contributes to the accumulation of triglycerides in hepatocytes, a hallmark of NAFLD. Insulin resistance results in a heightened flow of free fatty acids to the liver, which contributes to the buildup of fat and steatosis in the liver. This process is exacerbated by the pro-inflammatory state associated with obesity, marked by altered adipokine profiles and elevated levels of circulating cytokines, which further impair liver function and promote the progression of NAFLD. The altered secretion of adipokines such as leptin and adiponectin, along with an increase in pro-inflammatory cytokines like TNF-α and IL-6 due to visceral adiposity, plays a pivotal role in the progression of NAFLD [28, 29]. Specifically, the reduction in adiponectin levels associated with abdominal obesity contributes to heightened insulin resistance and liver inflammation, further exacerbating NAFLD [30]. Besides, the lipotoxicity associated with excessive fatty acid accumulation in the liver contributes to cellular injury and inflammation, paving the way for NAFLD progression [31]. The gut-liver axis also plays a role, where changes in gut permeability and microbiota in obesity can lead to increased exposure of the liver to gut-derived toxins, further exacerbating liver inflammation and steatosis [32, 33]. Genetic and epigenetic factors may modulate individual susceptibility to NAFLD in the context of obesity, highlighting the complex interplay of genetic predispositions and environmental factors in the disease's pathogenesis [34]. WC is a direct measure of abdominal or visceral fat, which is metabolically active and has a more significant impact on metabolic health than subcutaneous fat. Visceral adiposity is closely linked to insulin resistance, systemic inflammation, and altered secretion of adipokines [35], all of which contribute to the development and progression of NAFLD. In our study, increased WC is associated with higher NAFLD risk highlighting the importance of central obesity in NAFLD pathogenesis. Our results are corroborated by the robust association between increased WC and higher NAFLD risk, similar to the results presented by Kuang et al., who noted that abdominal obesity was a stronger predictor of NAFLD than general obesity due to its association with adverse metabolic profiles [36]. Furthermore, our analysis using RCS indicated a non-linear relationship between WC and NAFLD risk, with a steep increase at higher WC levels. This finding is particularly significant as it suggests that interventions aimed at reducing WC could potentially lower the risk of developing NAFLD.

The distinct impact of obesity types on NAFLD risk, particularly the significant effect of compound obesity, highlights the critical need for a detailed understanding of obesity's role in NAFLD pathologies. This understanding is crucial, given the complex interplay between metabolic dysfunctions associated with obesity and NAFLD progression. Our subgroup analyses revealed that the increased NAFLD risk associated with obesity patterns remained consistent across various demographic and clinical subgroups, indicating the universal relevance of obesity management in NAFLD prevention strategies. However, the lack of association between abdominal obesity and NAFLD in individuals with existing CVD, diabetes, or hypertension may point to a complex interplay between these conditions and liver disease, warranting further investigation. The strong association between WC increments and higher NAFLD risk, seen across all models, emphasizes the clinical utility of WC as a simple yet effective tool for NAFLD risk stratification. The observed non-linear positive relationship, especially the sharp increase in risk higher WC quantiles seen in RCS analysis, underlines the steep rise in NAFLD risk associated with central obesity. This is further substantiated by the ROC analysis, which identified an optimal WC cutoff for NAFLD risk, providing a practical reference for clinical assessments.

The combined assessment of BMI and WC offers a more comprehensive evaluation of NAFLD risk. Individuals with both high BMI and WC are at a particularly high risk of developing NAFLD, reflecting the combined effects of general and abdominal obesity. This group may experience more severe insulin resistance, systemic inflammation, and lipotoxicity, which can accelerate the progression of NAFLD to more advanced stages, including non-alcoholic steatohepatitis (NASH), fibrosis, and cirrhosis [37, 38]. Therefore, controlling BMI and WC with lifestyle changes, including changes in diet, more exercise, and losing weight, is essential for the prevention and treatment of NAFLD. These interventions can improve metabolic health, reduce liver fat, and help prevent the progression of liver disease.

However, our study has certain limitations. Firstly, the cross-sectional nature of our study limits our ability to definitively determine cause and effect. Further research is needed to establish the causal connections and underlying mechanisms. Secondly, diagnosis of NAFLD was based on ultrasonography rather than liver biopsy, which might lead to misclassification and an underestimation of its prevalence. Despite this, ultrasound is a more feasible option for NAFLD screening and is recommended by the World Federation for Ultrasound in Medicine and Biology [39].

## Conclusion

Our study provides significant insights into the associations between different patterns of obesity and the risk of NAFLD. The findings emphasize the need for targeted interventions focusing on weight management and metabolic health to prevent NAFLD, especially among those with compound obesity. Future longitudinal studies are needed to further elucidate the causal relationships and mechanisms underlying these associations.

### Supplementary Information

Supplementary Material 1.

## Data Availability

No datasets were generated or analysed during the current study.
