# Supplementary material for: Association between different obesity patterns and the risk of NAFLD detected by transient elastography: a cross-sectional study
Source: BMC Gastroenterol. 2024 Jul 10;24:221. doi: 10.1186/s12876-024-03303-x (PMC11238456; doi:10.1186/s12876-024-03303-x)
Supplement: Supplementary file 1 — Supplementary Material 1. [file 12876_2024_3303_MOESM1_ESM.docx]

**Table S1** Multivariate logistic regression of associations between different patterns of obesity and NAFLD risk after excluding individuals with history of malignancy

|  | Model I | Model II | Model III |
| --- | --- | --- | --- |
| Exposure | OR (95%CI), P | OR (95%CI), P | OR (95%CI), P |
| Normal Weight | 1(Reference) | 1(Reference) | 1(Reference) |
| General Obesity | 9.80 (5.59, 17.20) <0.0001 | 11.15 (6.23, 19.94) <0.0001 | 6.59 (3.57, 12.17) <0.0001 |
| Overweight | 4.02 (3.41, 4.73) <0.0001 | 3.87 (3.26, 4.59) <0.0001 | 2.67 (2.22, 3.22) <0.0001 |
| Abdominal Obesity | 3.36 (2.29, 4.92) <0.0001 | 3.80 (2.54, 5.68) <0.0001 | 3.01 (1.95, 4.64) <0.0001 |
| Compound Obesity | 13.55 (11.42, 16.09) <0.0001 | 17.71 (14.69, 21.36) <0.0001 | 8.95 (7.25, 11.06) <0.0001 |

Model I adjust for: None

Model II adjust for: gender, age, race, education level;

Model III adjust for: gender, age, race, education level, ALT, AST, total bilirubin, ALP, GGT, uric acid, total cholesterol, HDL cholesterol, CVD, smoking status, diabetes, hypertension, statins, antihypertensive drugs, and antidiabetic agents.
